# Pneumonia and Broncho-Pneumonia

**Published:** 1898-06-04

**Authors:** 


### PNEUMONIA AND BRONCHO-PNEUMONIA.

It has been commonly considered that the lobular pneumonia so common among infants and the lobar pneumonia met with in adults are two distinct diseases. Dr. Samuel West has recently shown, however, that the distinctions between these diseases will not hold with anything like the preciseness they should if the two diseases were really as different as they have been imagined to be. So far as post-mortem appearances go no doubt the pneumonia from which infants die is mostly scattered and lobular, and that which kills adults massive and lobar. But if we investigate the matter as a question of bacteriology, Dr. West shows that many of the lobular pneumonias of infancy exhibit the pneumococcus; while if we observe cases of lobular pneumonia clinically we shall see that many among them are clearly not consecutive to bronchitis, but have the same sudden onset, high temperature, and rapid defervescence as occur in the lobar pneumonia of adults, and Dr. West asserts that if we place these cases apart as a separate class, and examine them bacteriologically, we shall find the pneumococcus as frequently in them as we do in the lobar pneumonia of adults. This result is extremely interesting. We have two main forms of pneumonia to deal with. The division, however, must no longer be into (*a*) lobular or patchy pneumonia, and (*b*) lobar or massive pneumonia, but into (1) secondary broncho-pneumonia, after bronchitis, measles, whooping-cough, diphtheria, &c., and (2) croupous pneumonia, which although in the adult massive is in the child lobular. Of the secondary broncho-pneumonia which follows bronchitis or other acute febrile ailments we have two forms, both of which are lobular. In one there is a gradual onset and a tendency to relapse. Fever is hectic, markedly remittent, of long duration and gradual fall. This is due to a streptococcal infection. In the other there is a gradual onset with sudden aggravation of symptoms, but in other respects it resembles the form just described. Here the infection is due to streptococcus, &c., associated probably with pneumococcus. Croupous pneumonia also occurs in two forms—one the ordinary lobar form, as in the adult; the other as a primary broncho-pneumonia, with sudden onset, no tendency to relapse; fever high, not markedly remittent, of short duration, with sudden fall, the microbe in each case being the pneumococcus. As Dr. West says: "The proper scientific place for the primary forms of broncho-pneumonia is not with broncho-pneumonia at all as it is commonly understood, but with croupous pneumonia."

